# A Scoping Literature Review of Rural Institutional Elder Care

**DOI:** 10.3390/ijerph191610319

**Published:** 2022-08-19

**Authors:** Mingyang Li, Yibin Ao, Shulin Deng, Panyu Peng, Shuangzhou Chen, Tong Wang, Igor Martek, Homa Bahmani

**Affiliations:** 1 College of Management Science, Chengdu University of Technology, Chengdu 610059, China; 2 College of Environment and Civil Engineering, Chengdu University of Technology, Chengdu 610059, China; 3 Department of Social Work and Social Administration, Faculty of Social Sciences, The University of Hong Kong, Hong Kong, China; 4 Faculty of Architecture and Built Environment, Delft University of Technology, 2628 CD Delft, The Netherlands; 5 School of Architecture and Built Environment, Deakin University, Geelong 3220, Australia

**Keywords:** rural areas, institutional elder care, science mapping, scoping analysis, literature review

## Abstract

Under circumstances of pervasive global aging combined with weakened traditional family elder care, an incremental demand for institutional elder care is generated. This has led to a surge in research regarding institutional elder care. Rural residents’ institutional elder care is receiving more attention as a major theme in social sciences and humanities research. Based on 94 articles related to rural institutional elder care, this study identified the most influential articles, journals and countries in rural institutional elder care research since 1995. This was done using science mapping methods through a three-step workflow consisting of bibliometric retrieval, scoping analysis and qualitative discussion. Keywords revealed five research mainstreams in this field: (1) the cognition and mental state of aged populations, (2) the nursing quality and service supply of aged care institutions, (3) the aged care management systems’ establishment and improvements, (4) the risk factors of admission and discharge of aged care institutions, and (5) deathbed matters regarding the aged population. A qualitative discussion is also provided for 39 urban and rural comparative research papers and 55 pure rural research papers, summarizing the current research progress status regarding institutional elder care systems in rural areas. Gaps within existing research are also identified to indicate future research trends (such as the multi-dimensional and in-depth comparative research on institutional elder care, new rural institutional elder care model and technology, and correlative policy planning and development), which provides a multi-disciplinary guide for future research.

## 1. Introduction

The rapidly aging population combined with the weakening of traditional family aged care support [1,2,3,4] has received much attention in institutional elder care research which is reflected by exponential growth in relevant academic literature in recent years [5]. Scholars have made fruitful advances in the research theme of institutional elder care. Nevertheless, despite this progress, there is a lack of similar advances regarding institutional elder care in rural areas. The matter is significant as there are presently 3.4 billion rural residents confronted with institutional elder care challenges [6]. Therefore, a comprehensive scoping review of rural institutional elder care research would be a timely addition to the body of knowledge for stakeholders, such as rural planners, researchers, and government bodies, to alleviate the plight of aging rural populations and further narrow the urban–rural gap. Considering this background, this study attempts to construct a bibliometric atlas of related disciplines and research fields impacting rural institutional elder care through science mapping, bibliometric search, and scoping analysis [7]. This approach is superior in reducing subjective bias while identifying current research themes and journal sources, along with the mapping of major countries and authors delving into rural institutional elder care research. Such an approach provides a research scope which forms a foundation for scholars concerned with rural regional development, in to pursue this further and to chart fresh research ventures into neglected concerns.

The contribution of this study to the research field of rural institutional elder care is as follows: (1) The selection method of the literature samples covered in this paper is relatively comprehensive, and the high-frequency keywords related to rural institutional elder care extracted from the literature samples can reflect current research topics; (2) This paper presents high-impact articles, journals, countries, and authors related to rural institutional elder care; (3) This research explores the research evolution in the field of rural institutional elder care by using a scoping analysis method, and discusses the gaps within existing research, to identify future research topics. It should be noted that this review is a summary of the global research on rural institutional elder care, and in order to ensure the comprehensiveness of the review, it is not based on any country’s definition standard of “rural” and “urban”. The division of “rural” and “urban” in this review follows the division criteria in the original literature samples. In other words, this review distinguishes between “rural” and “urban” based on the results of the search formula TS = (“rural” OR “village“) and the specific content of the literature sample. Meanwhile, among the 94 literature samples, 39 comparative studies of urban and rural institutional elder care were retained, assuring enough relevant rural sample for conclusions.

The remainder of this paper is organized as follows. The following section describes the overall research methods of science mapping and qualitative discussion. The third section shows the visualized scientific atlas and the results of the scoping analysis. The fourth section expands on the above analysis results and discusses the research gaps and trends in this field. The final section summarizes current research as identified and described in this review.

## 2. Methodology

**Figure 1:**
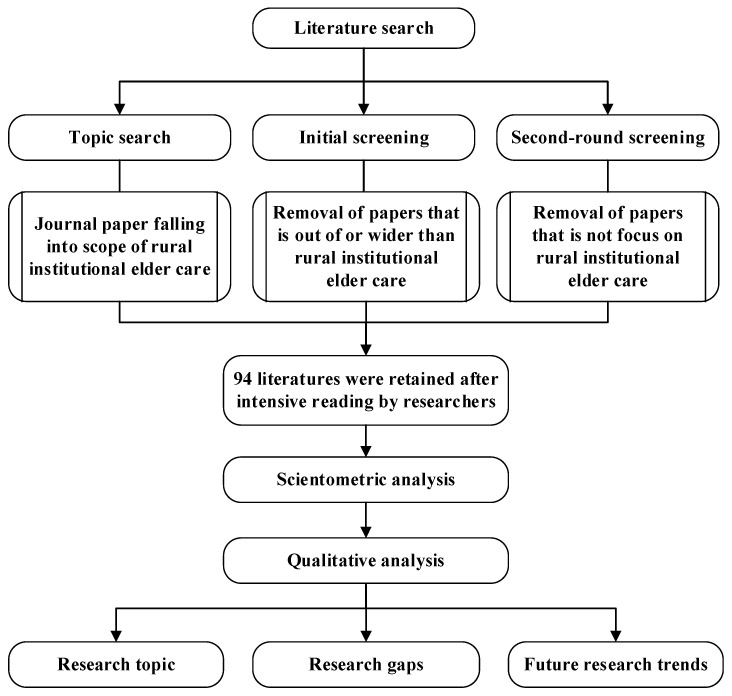
Flow chart of scoping analysis of literature on rural institutional elder care.

Introduced by Pritchard [8], bibliometric review and analysis are referred to as a quantitative analysis of patterns and boundaries to provide insights into the research of a specific field. This study adopts this view and uses a bibliometric review method to evaluate the research results of rural institutional elder care as published by scholars on the Web of Science Core Collection, which consists of SCIE (Science Citation Index-Expanded), SSCI (Social Sciences Citation Index), A&HCI (Arts & Humanities Citation Index), ESCI (Emerging Sources Citation Index), CCR-EXPANDED (Current Chemical Reactions), IC (Index Chemicus). The overall research workflow is shown in Figure 1, which explicitly encompasses three parts: literature retrieval and screening, scoping analysis, and qualitative discussion.

### 2.1. Bibliometric Search

**Figure 2:**
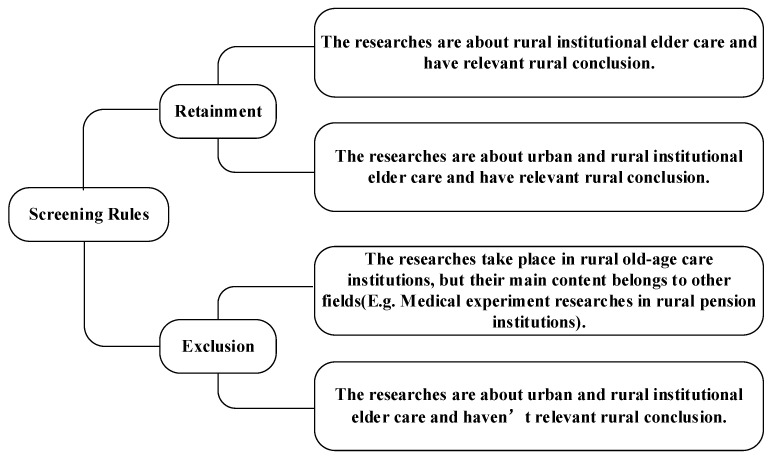
The rules of screening samples.

The Web of Science combines a traditional citation index with advanced Web technology and multiple and unique functions that accurately retrieve targeted literature. Moreover, it contains comprehensive literature information [9]. As such, the Web of Science Core Collection was used for conducting the literature retrieval. The search rule used is TS = (“rural” OR “village”) AND TS = (“old” OR “aged” OR “elderly”) AND TS = (“nursing home” OR “institutional pension” OR “pension institution” OR “nursing facility” OR “sanatorium” OR “Welfare home” OR “bead house” OR “geracomium”), resulting in 437 records being retrieved. Thus, 437 retrieved documents were used as the basic database of the literature review. Subsequently, a browse of the titles and abstracts led to a culling of 277 articles as being unrelated to rural institutional elder care. On a detailed browse of the remaining 160 publications, a further 66 were excluded as off-topic. In these cases, while the articles may mention rural institutional elder care, reference was only incidental without focusing on the research theme, such as *Pain deterioration within 1 year predicts future decline of walking ability: A 7-year prospective observational study of elderly female patients with knee osteoarthritis living in a rural district*; *Multidrug-resistant organism infections in US nursing homes: A national study of prevalence, onset, and transmission across care settings* and *1 October 2010–31 December 2011, Prevalence of methicillin-resistant Staphylococcus aureus (MRSA) in patients in long-term care in hospitals, rehabilitation centers and nursing homes of a rural district in Germany* [10,11,12]; Other excluded articles did not offer research conclusions regarding rural institutional elder care, such as *Telerehabilitation for older people using off-the-shelf applications: Acceptability and feasibility* and *Assessing end-of-life preferences for advanced dementia in rural patients using an educational video: A randomized controlled trial* [13,14]. Further screening parameters are shown in Figure 2. Ultimately, the number of documents deemed suitable for further analysis in this study amounted to 94.

### 2.2. Science Mapping

VOSviewer, a text-mining tool developed by Van Eck and Waltman [15], was adopted in this study for analyzing and visualizing bibliometric networks. Based on the text mining features, VOSviewer can be used to analyze research keywords assisted by visualization [16]. It also provides quantitative metrics (e.g., citation-based measurement) to evaluate the impact of research keywords, journals, scholars, documents, or literature sources [17]. The following goals were pursued through science mapping: (1) To load the literature samples from the Web of Science Core Collection; (2) To visualize, calculate and analyze the influence of mainstream journals, scholars, papers and countries in the field of rural institutional elder care; (3) To study the main research keywords in the field of rural institutional elder care and identify their relationships.

### 2.3. Qualitative Discussion

Qualitative discussion is the final step required following a bibliometric search and scoping analysis. Qualitative research is always helpful to provide readers with more in-depth and useful insights [18,19]. An in-depth qualitative discussion of mainstream research topics provides important outcomes for readers of interest, including summarizing the main research results in the current rural institutional elder care field, identifying the limitations or gaps in current research, and proposing future research directions.

## 3. Results of Scoping Analysis

### 3.1. Overview of Literature Samples

**Figure 3:**
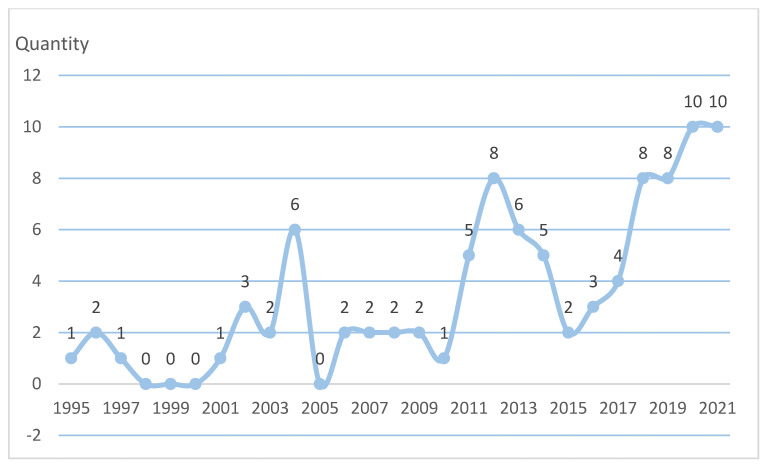
Yearly publications from 1995 to 2021.

Generally speaking, scholars pay limited attention to rural institutional elder care. From Figure 3, it can be found that the number of related articles published before 2003 was not large, while the number of related articles published after 2003 increased somewhat. While the number of published articles fluctuated greatly, an overall increasing trend was demonstrated. (The time point of literature retrieval in this study was 1 January 2022).

### 3.2. Science Mapping of Journal Sources

**Figure 4:**
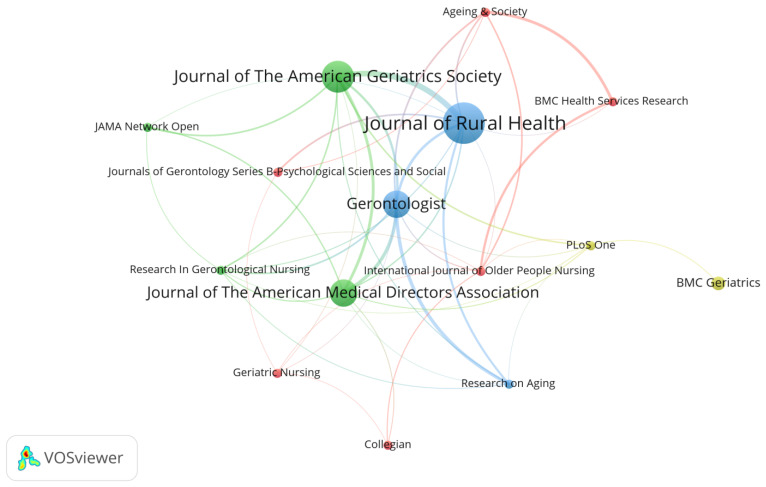
Map of mainstream journals in the field of rural institutional elder care.

This study systematically analyzes the source journals of 94 research articles on rural institutional elder care. The results are shown in Figure 4. Setting the minimum number of published papers to two and the minimum number of cited papers to 10 in VOSviewer gave rise to 15 out of 57 journals reaching this threshold. Figure 4 shows the clusters of journal sources with their relationships visualized by connection lines. More detailed information on the journal source is supplemented in Table 1.

In Figure 4, the font and node size visually and proportionally represent the number of publications of the given journals. At the same time, different colors and the connecting lines indicate the closeness of mutual citation between journals [15]. Citation is one of the main indicators for measuring the influence of academic works, and the use of direct citations is a commonly accepted measure in academia for identifying influential studies in a domain [20]. Table 1 summarizes the number of publications, the total citations, the average citations of each journal, and the average normalized citations. These four indicators collectively indicate the influence of journals and quantify the impact of journals in the field of rural institutional elder care. Figure 4 and Table 1 display the most influential journals. Particularly, *Journal of Rural Health* is found with the highest literature output, followed by *Journal of the American Geriatrics Society,* with both the second highest literature output and average citation times. From the perspective of average normalized citation, journals such as *Collegian* and *JAMA Network Open* are rated with the highest annual average influence.

### 3.3. Co-Authorship Analysis

**Figure 5:**
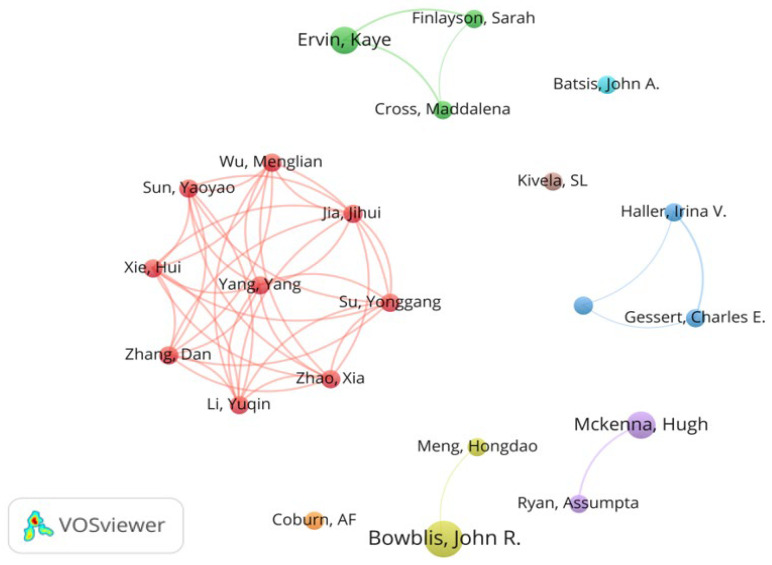
Map of co-authorship in the field of rural institutional elder care. Reference: [21,22,23,24,25,26,27,28,29,30,31,32,33,34,35,36,37,38,39,40,41,42,43]. Note: The reference citation number herein is the reference citation number in the text.

The minimum number of documents of an author and the minimum number of citations of an author set in VOSviewer are 2 and 15, respectively. Of the total 357 authors in the literature sample, 22 met the selection criteria. Literature influence was assessed using Norm. Citations. As shown in Figure 5, the series of studies conducted by Bowblis and John have made important contributions in this field. Table 2 lists further details of co-authorship analysis.

Chinese researchers indicated by the red clusters in the co-authorship map contributed the highest value of Norm. These citations focused on exploring the relationship between social support and mental health and health-related quality of life in rural nursing homes in China and the gender differences in institutional care choices. For example, Zhang, et al. [21] constructed a multiple regression model to explore the influencing factors of suicidal ideation and found that suicide ideation is indirectly affected by self-esteem and loneliness but directly affected by depression and despair. Wu, et al. [22] used resilience as an intermediary to show the indirect impact of social support on health-related quality of life. Qian, et al. [44] found gender differences in the willingness of single elderly people to use institutional care in rural China, where single rural women are more reluctant to accept institutional care than single rural men.

### 3.4. Countries Active in Rural Institutional Elder Care Research

**Figure 6:**
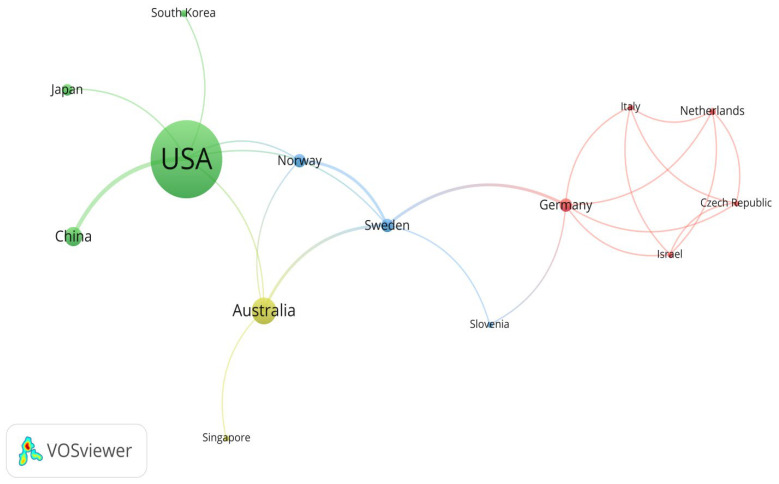
Map of countries actively engaged in research on the rural institutional elder care.

This study identified those countries active in rural institutional elder care research, with the United States, Australia, and China standing out. Using VOSviewer, these countries’ contribution to rural institutional elder care research is identified and evaluated. When drawing the map of countries conducting research in rural institutional elder care research, the minimum number of documents of a country and the minimum number of citations of a country were both set to 1. Finally, 23 out of 25 countries met the conditions. Figure 6 shows 14 countries and their mutual relationships among 23 countries that are actively engaged in this research field, with additional information on other countries with relatively weak ties supplemented in Table 3.

**Figure 7:**
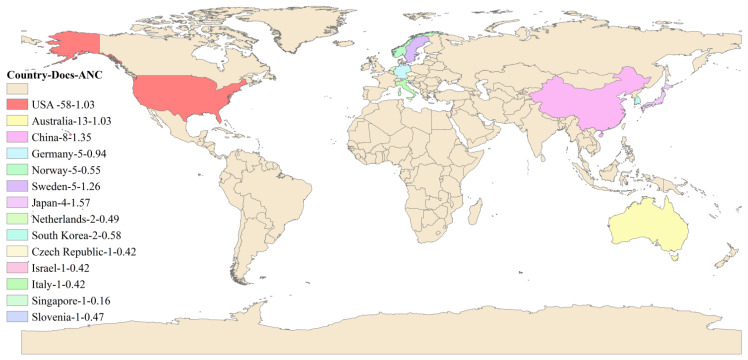
The geographical map of countries actively engaged in the research topic.

Figure 7 is drawn according to the geographical location, the number of published articles, and the average normalized citation of 14 countries that are actively engaged in rural institutional elder care research. Researchers for developed countries such as the USA, Australia, Germany, and Norway devote significant efforts to researching rural institutional elder care. However, an emerging trend has also been found in other developing countries like China, with increasing enthusiasm in investigating rural institutional elder care, probably due to their ever-growing elderly population.

Table 3 provides more quantitative measurement data, including the number of publications, mutual citations, the average publication year, the total number of citations, the average number of citations, and the average number of normalized citations. According to Table 3, the USA, Australia and China have both the most significant number of publications and the most extensive citation according to the average number of normalized citations, and Portugal, Japan, Switzerland, and China show the strongest influence in this research field.

### 3.5. Co-Occurrence of Keywords

**Figure 8:**
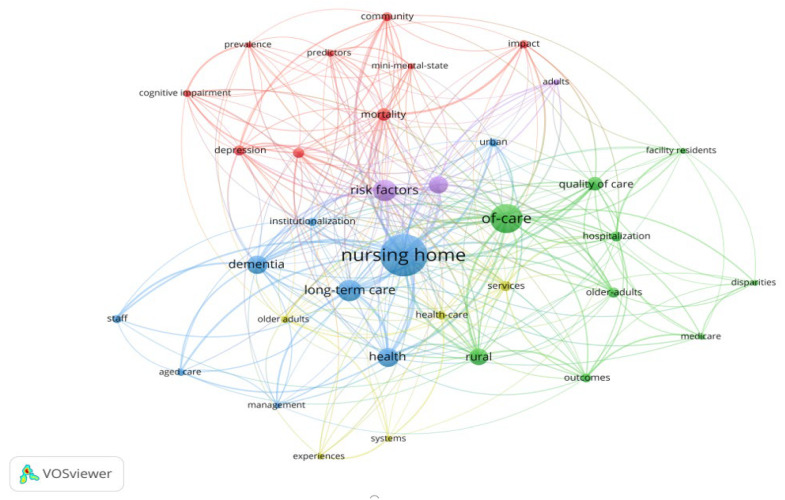
Map of co-occurrence of keywords in the rural institutional elder care research.

Keywords spotlight the main topic content of studies and depict the themes that have been focused on within a given domain [45]. A network of keywords displays the knowledge inter-relationships and intellectual organization of research themes [16]. Using “All keywords” and “Fractional counting” in VOSviewer, the minimum occurrence of a keyword was set to four. Initially, 45 of the 522 keywords reached the threshold. Afterwards, two rounds of keywords screening were conducted to select more specific keywords for further analysis. In the first round, through detailed scrutinization, some general items with limited semantic value, such as “residents”, “people”, “cost”, “end”, and “access” were removed from the initially scoping 50 keywords. In the second round, some other keywords with the same semantics were combined, such as “nursing homes” and “nursing home”, “older-people”, “older-adults”, and “long-term-care” and “long-term care”. Finally, a total of 35 keywords were selected, as shown in Figure 8.

Based on the connection strength between keywords, VOSviewer divides 35 keywords into five clusters with different colors for distinction: red, green, blue, purple, and yellow (see Figure 8). It can be seen that keywords such as “cognitive impairment”, “depression”, and “mini-mental-state” belong to the red cluster, which represents aspects related to the cognitive situation and mental state of aged people. The green cluster includes keywords related to nursing quality and service supply in old-age care institutions, such as “of-care” and “quality of care”. The blue cluster consists of some keywords related to the establishment of old-age care institutions’ management systems, such as “Long-term care”, “management”, and “institutionalization”. The purple cluster comprises some keywords in correlation with risk factors of admission and discharge of aged care institutions, such as “risk factors”. Finally, some keywords related to healthy institutional elder care and deathbed matters of aged population are included in the yellow cluster, such as “health-care” and “services”. Similarly, keywords of different clusters are closely related, such as “hospitalization”, “health-care”, and “mini-mental-state”. Generally speaking, the mainstream research keywords of institutional elder care in rural areas can be classified according to these five different clusters, namely (1) the cognition and mental state of the aged population, (2) the nursing quality and service supply of aged care institutions, (3) the aged care management systems’ establishment and improvement, (4) the risk factors of admission and discharge of aged care institutions, and (5) the healthy institutional elder care and deathbed matters of the aged population.

Table 4 summarizes the quantitative measurement of more keywords. According to Ave. Norm. Citation, the keyword “cognitive impairment” has attracted substantial attention in rural institutional elder care research. The average publication year indicates the recentness of a keyword in the field of rural institutional elder care. For example, around 2011, most research focused on the psychometrics of aged people peaked, indicating that this topic was slightly out-of-date. In contrast, papers related to the comparative study of urban and rural institutional elder care were published only in around 2019 or later. These emerging topics have attracted researchers’ attention only in recent years and are more likely to pose a future research direction.

## 4. Discussion

Of the 94 identified research studies on rural institutional elder care, 39 offer relevant research conclusions based on comparative analyses between urban and rural institutional elder care, while the remaining 55 provide a more in-depth investigation specific to rural institutional elder care. According to the five mainstream research topics presented in the map of co-occurrence of keywords, studies on rural institutional elder care are classified.

### 4.1. Comparative Studies of Urban and Rural Institutional Elder Care

When exploring the present situation of institutional elder care in rural areas from urban–rural comparative studies, a series of similarities and differences between rural and urban institutional elder care are identified. To provide clarity for readers, the primary trend of urban–rural comparative studies is also discussed individually from the five research mainstreams identified from the keyword analysis, including (1) the cognition and mental state of the aged population, (2) the nursing quality and service supply of aged care institutions, (3) aged care management systems’ establishment and improvement, (4) the risk factors of admission and discharge of aged care institutions, and (5) healthy institutional elder care and deathbed matters of the aged population.

In the comparative studies of rural and urban institutional elder care on the cognition and mental state of the aged population, the happiness, satisfaction, depression, and cognitive level of residents and related personnel in rural old-age care institutions are the main research objects. Generally speaking, the happiness of the elderly in rural old-age care institutions is lower than that found in urban old-age care institutions [46]. The satisfaction of elderly relatives in rural old-age care institutions is also lower than that in urban old-age care institutions [47]. The elderly in rural old-age care institutions have a higher suicide tendency, more cognitive barriers and problematic behaviors than those in urban old-age care institutions [48,49]. However, some studies comparing urban and rural residents show that rural residents are in a better state concerning dementia behavior and psychological symptoms [50]. The prominent medical gap between urban and rural areas in the field of cognition and psychotherapy makes the probability of correct diagnosis and related treatment of mental illnesses such as depression among the elderly in rural old-age care institutions lower than that in urban old-age care institutions [51,52]. At the same time, the difference in benefits of medical policies between urban and rural areas makes it less likely for the elderly with dementia in rural areas to be placed in nursing homes or particular care institutions and receive less formal support [53].

In the meantime, from the comparative studies of rural and urban institutional elder care on the nursing quality and service supply of aged care institutions, it is generally believed that the level of nursing quality and service supply in old-age care institutions in urban areas is higher than in rural areas [54]. This fact provides a rational explanation for a large proportion of urban-rural gaps in various aspects, such as facility characteristics, funding sources, and implementation of medical and health care policies [23,24,25,55,56,57]. To remedy the deficiency of rural institutional elder care in nursing quality and service supply, the retention rate of community family doctors for rural elderly is suggested to be higher than that for urban elderly in the early stages of entering long-term care institutions [58]. However, life improvement and survival rates of rural elderly are still far from satisfaction compared with urban elderly [59,60].

Likewise, for rural and urban institutional elder care comparison concerning the elderly care management systems’ establishment and improvement, the main research focus lies in the characteristics and recruitment paths of certified nursing assistants. Although both the staff of nursing homes in urban and rural areas rely heavily on relatives and friends in order to obtain recruitment opportunities, rural nursing staff are more likely to receive training in nursing institutions than those in urban nursing homes that might be trained by community colleges or websites [61].

Furthermore, the differences between admission and discharge status of urban and rural residents and the exploration of related influencing factors remain a main focus. In the research on the status quo of admission to nursing homes, in general, the rural elderly tend to care for nursing homes better than the urban elderly [26,62,63,64]. The rehospitalization rates of rural elderly are also higher [65]. This difference may be attributed to the supply of beds in nursing homes [62] and the specific community context factors [64,66,67]. Under certain conditions, opposite research results may also arise. For example, the rural elderly enjoying a good community environment are less likely to choose nursing home care than the urban elderly [27,68], and their rehospitalization rate is also lower [69]. Regarding the status quo of discharge from aged care institutions, the emergency transfer rate of rural nursing institutions is lower than that of urban nursing institutions due to their limited traffic and medical diagnosis level [28,70]. Even worse, the overall success rate of community discharge of rural elderly is considerably lower than that of urban elderly [71].

In the comparative studies of rural and urban institutional elder care on the healthy institutional elder care and deathbed matters of the aged population. There are two main emphases, one health and disease, and the other end-of-life decisions and care. In addition, in the contrastive research of health and diseases of the elderly between urban and rural institutional elder care, on the one hand, the significant regional differences in the level and quality of health services of residents in nursing homes between urban and rural areas are confirmed across many quality indicators [72]. On the other hand, hip fracture and pain management are high-frequency research sites for common diseases of institutional elder residents [73,74]. For end-of-life decision-making and end-of-life care between rural and urban areas, the differences between rural and urban institutional elder care are also confirmed [75,76]. Likewise, urban and rural old-age care institutions’ end-of-life quality measures for residents’ in-hospital death and hospice referral before death, the rural nursing service institutions are clearly worse than the urban nursing service institutions [29]. Comparing the difference between the decision making and the use of advance directives of residents in nursing homes, residents from urban areas are more likely to make their own decisions on medical care, treatment, financial affairs, and legal affairs at the beginning of their stay in nursing homes. However, residents in rural areas are more likely to give advanced instructions and “do not resuscitate” orders [77]. Residents of rural nursing homes hospitalized in the last 90 days of their lives are more likely to leave living wills than urban residents [78].

### 4.2. Studies of Rural Institutional Elder Care

Studies on rural institutional elder care are more specific in scope than comparative studies. Certain characteristics unique to the rural setting can be found. To provide readers with structured and systematic insights, the discussion is also unfolded from the five research mainstreams identified from the keyword analysis, namely (1) the cognition and mental state of the aged population, (2) the nursing quality and service supply of aged care institutions, (3) the aged care management systems’ establishment and improvement, (4) the risk factors of admission and discharge of aged care institutions, and (5) the healthy institutional elder care and deathbed matters of the aged population.

In rural institutional elder care studies on the cognition and mental state of the aged population, scholars have described the cognition and mental state of rural institutional elder care-related groups, such as residents, family members and staff [30,79,80]. For example, Johnston, et al. [81] found that, in the measurement of depression of the elderly in rural care institutions, it is difficult to identify their depression because of the inconsistency among residents, nursing staff and screening tools. Health professionals concerned with depression in the elderly should be aware of the differences among various identification sources. By increasing social support and improving physical health, depression and despair can be avoided to effectively prevent and intervene in the suicidal behavior of elderly residents in rural nursing homes [31]. Communal life in rural old-age care institutions can alleviate depression and improve the life satisfaction of the elderly [82]. However, in recent years, the existing social networks of residents in rural old-age care institutions have been significantly reduced in number and diversity, posing the risk of social isolation [21,83,84]. As a promising solution, long-distance mental consultation services can alleviate the problem of mental illness in rural nursing homes to some extent [85], with apparent advantages in saving both time and money [86]. In addition, due to the low cognitive function of dementia patients [87], the care pathway for dementia in nursing homes is also under exploration; however, the so-called Liverpool care pathway is considered unadaptable to dementia care in rural nursing homes [88].

For the nursing quality and service supply of aged care institutions, the influence of facilities and service conditions of rural nursing homes on the quality of life, physical health, mental health, and social relations of the elderly has been confirmed [89]. For the rural elderly, more dependence on Medicaid for long-term care has also been found [32]. Some studies show that rural residents are more likely to live in facilities without certification or special nursing programs, which may reduce their chances of receiving quality nursing care. Therefore, suggestions have been proposed to strengthen the policy effect of medical insurance payment methods, improve the certification status of rural facilities, and provide special nursing plans for reducing variability in nursing quality [89], such as pain management activities [90,91,92], computerized decision support systems (CDSS) [93], management planning [94], virtual reality-based progressive resistance training [95], case management [34], and obesity management [35].

For the rural aged care management systems’ establishment and improvement, research topics can be roughly divided into two streams, staff management and institution management. In terms of staff management, there are a range of problems leading to the ineffective implementation of management systems. Some of these shortcomings are as follows: inadequate consideration of family members’ opinions by management and staff [96], lack of comprehensive nursing plans [97], limited resources [96], and insufficient continuous education for staff [98]. Due to information asymmetry and other factors in the management of old-age residents, there are inconsistencies in the practices of nursing staff during the initial transition period when the elderly enter long-term care institutions [99]. This shortcoming can be minimized by continuous and spontaneous staff collaboration [100]. However, the limitation of working hours remains a hurdle to achieving better results [36,37]. Studies have long been calling for the development of caring partnerships between nursing staff and families [38], embracing of advanced nursing plans [101], introducing self-management intelligent nursing home frameworks [102], meeting nursing staff’s educational needs [98], and avoiding violence among residents [103,104].

In studies of rural institutional elder care on the risk factors of admission and discharge of aged care institutions, related factor analysis has always been a research priority. A high social capital level influences family members’ familiarity with nursing homes in rural communities [76]. In turn, familiarity also affects the choice of nursing homes, the timing of placing the elderly, and the response of family caregivers [39]. When choosing an appropriate nursing home, proximity to community and family members is an important criterion [105]. The imbalance of self-care function (physical, social, mental, and daily living ability) of the elderly may increase their chances of staying in nursing homes [106,107,108]. At the same time, older adults living in different old-age care institutions show heterogeneity in functional evaluation [109]. However, some studies have indicated that, if rural old-age residents can delay functional decline, their discharge plan can possibly be accelerated [40].

Regarding healthy rural institutional elder care and deathbed matters regarding the aged population, two main themes are identified: health and disease and end-of-life decision and care. In the field of health and diseases, the main concerns are adverse drug reactions in old-age care institutions [110], microbial environment differences and infection paths [111,112,113], the efficacy of medications and the popularity of various drugs [41,114], and risk factors of fall injury [42]. In addition, some research institutes try to combine social factors with institutional care to explore the health of the elderly. For example, Wu, et al. [22] use resilience as an intermediary to prove the indirect impact of social support on health-related quality of life. In the field of end-of-life decision-making and care, some studies have found that determining end-of-life planning will increase the probability of choosing institutions for old-age care, directly demonstrating the importance of care acceptability in end-of-life planning [105]. In addition, the records of preferences for dying showed that 64% of rural residents asked for a natural death or no cardiopulmonary resuscitation. However, medical interventions (including drugs, fluids or nutrition) were more preferred than cardiopulmonary resuscitation [101]. With the rapid development of science and technology, nursing home care driven by information and communication technology (ICT) can make residents continue on in nursing homes for a longer period in contrast to staying in hospitals [115].

### 4.3. Research Gaps and Trends

**Figure 9:**
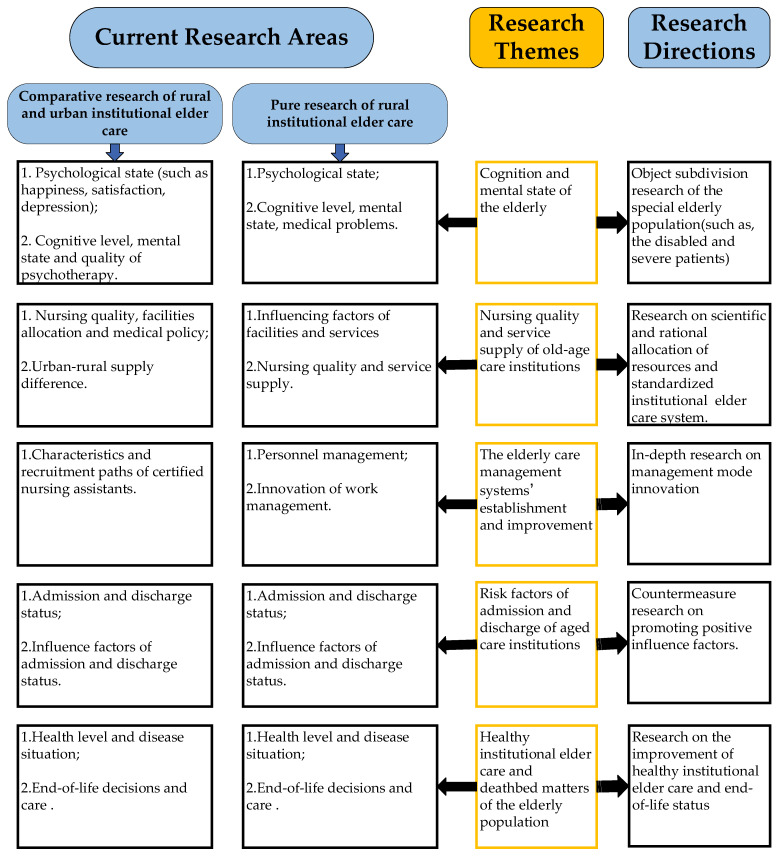
Framework linking current research topics to future research directions.

Although the number of published research studies on institutional elder care in rural areas is relatively small, significant research gaps can be summarized by analyzing the existing literature to inform related stakeholders of future research directions. Based on the discussion of mainstream research topics and gaps, the framework of near-future directions in rural nursing homes is proposed and shown in Figure 9.

#### 4.3.1. Object Subdivision Research Can Bring Wider and Deeper Value

Nowadays, comparative studies between urban and rural institutional elder care are an evident priority, where differences between urban and rural depressed and demented elderly are among the most commonly adopted research themes [43,51,53,78,116]. However, there are few studies regarding other elderly groups, such as the disabled or the seriously sick, suggesting that scholars conduct further penetrative explorations for the building of more scientific rural institutional elder care models.

#### 4.3.2. Forward-Looking Attention to Scientific and Rational Resource Allocation and Standardized Institutional Elder Care System

Compared with urban institutional elder care, the resource allocation of rural institutional elder care shows great room for improvement. Thus, scientific and rational resource allocation is needed for rural institutional elder care. Future research on staffing and capital investment is recommended. Furthermore, the rural institutional elder care system should be standardized, so that the innovation research of rural institutional elder care models is required for accumulation. In recent years, intelligent nursing homes [102], telemedicine [35,85,86], and other emerging topics have received more attention. Further research on new rural institutional elder care models assisted with emerging technologies will significantly help improve the life quality of rural institutional elder care residents and enhance their wellbeing.

#### 4.3.3. In-Depth Research on Management Mode Innovation Is Required

The essence of the nursing home is an institution, and its management mode is the core. According to the present research situation, the management mode of rural nursing homes is backward, so further research on management mode innovation is needed to promote the development of rural elder care.

#### 4.3.4. Policy Planning Needs-Related Policy Research as Foundation

Descriptive and empirical research on influence factors of the admission and discharge of nursing homes pave the way for countermeasure research. The significance of countermeasure research lies in the accumulation of policy planning. With a global trend of an aging population, many countries have attached great importance to planning and developing old-age care policies [117], suggesting further investigation of their suitability under complex dynamic conditions for tailoring ever more appropriate responsive policies and strategies [32]. Besides, in the future, when the government implements a new policy, it is still necessary to make a comparative study before and after the implementation of the policy.

#### 4.3.5. The Improvement of Healthy Institutional Elder Care and End-of-Life Status Is Appealed

Overall, the health level, illness condition, and end-of-life status of the elderly in rural old-age care institutions are worse than those in urban old-age care institutions. Furthermore, the improvement of healthy institutional elder care and end-of-life status is called for by society.

## 5. Conclusions

This study of rural institutional elder care follows a bibliometric research paradigm, employing scoping and qualitative analysis to find the current research scope and future directions. A total of 94 related articles are selected as samples for analysis with results listed as follows: (1) The influential journals that publish papers on rural institutional elder care include *Journal of Rural Health*, *Journal of the American Geriatrics Society*, *Collegian* and *JAMA Network Open*. (2) Keywords are determined by co-occurrence keyword analysis, and the mainstream research keywords of institutional elder care in rural areas can be classified into five aspects: the cognition and mental state of the aged population; the nursing quality and service supply of aged care institutions; aged care management systems; risk factors of admission and discharge of aged care institutions, and healthy institutional elder care and deathbed matters of the aged population. (3) Co-authorship analysis reflects the most frequently cited articles from Yang et al. who have the highest Norm. Citations, with their work mainly exploring the relationship between social support and mental health, and health-related quality of life in rural nursing homes in China, as well as the gender differences in institutional care choices. (4) The countries active in carrying out research on rural institutional elder care include the United States, Australia, and China, significantly contributing to this research field.

### 5.1. Practical Implications

According to the outcomes of scoping analysis, this study further identifies gaps within this research field. Consequently, a systematic framework for further research is proposed: (1) There are only a few multi-dimensional comparative studies, and in the future, more attention should be paid to rural institutional elder care comparisons between developed countries and developing countries, comparison of various indicators of institutional old-age care in urban and rural areas, and comparison between ordinary old-age groups and special old-age groups; (2) Compared with urban institutional elder care systems, rural institutional elder care models and technology have greater room for improvement, and both the market and the government should actively encourage innovative research on rural institutional elder care; (3) Studies on urban institutional elder care have offered many notable recommendations, but those addressing rural areas and their circumstances are limited, and the sustainable development of rural institutional elder care model remains absent. In order to identify developmental pathways and provide practical policy recommendations, further efforts to upgrade and revitalize rural institutional elder care are needed.

### 5.2. Limitation

This study focuses on the research progress of rural institutional elder care, with the research results limited by the scope of the research literature samples but not specifically listed and selected the definition of urban and rural research. Furthermore, this research sample is derived from Web of Science Core Collection only, which may limit the scope of the review. In defining the literature sample, the completeness is constrained by the keyword indicators on which the sample selection is predicated so future research could expand the search query. Moreover, only journal papers were selected, excluding minutes, letters, etc., which ordinarily offers limited insight, but this remains an assumption. This study only contains English literature, and does not analyze the research output published in other languages, which also may result in the omission of important references in related fields.

## Figures and Tables

**Table 1:** Quantitative measurements of journals publishing rural institutional elder care research.

Source	Number of Publications	Total Citations	Average Citations	Ave. Norm. Citation
Journal of Rural Health	9	131	14.56	0.66
Journal of The American Geriatrics Society	7	247	35.29	1.33
Gerontologist	6	84	14.00	1.05
Journal of The American Medical Directors Association	6	68	11.33	0.96
BMC Geriatrics	3	32	10.67	0.88
Ageing & Society	2	22	11.00	0.67
BMC Health Services Research	2	18	9.00	0.38
Collegian	2	42	21.00	1.71
Geriatric Nursing	2	15	7.50	0.53
International Journal of Older People Nursing	2	37	18.50	1.38
JAMA Network Open	2	21	10.50	1.67
Journals of Gerontology Series B-Psychological Sciences and Social	2	84	42.00	0.99
PLoS One	2	20	10.00	1.30
Research In Gerontological Nursing	2	17	8.50	0.49
Research on Aging	2	11	5.50	0.85

**Table 2:** Quantitative measurements of scholars in rural institutional elder care research.

Scholar	Documents	Citations	Avg. Pub. Year	Norm. Citations	Avg. Citations	Avg. Norm. Citations
Bowblis, John R. [23,24,27,28]	4	36	2019	3.31	9.00	0.83
Ervin, Kaye [34,36,37]	3	41	2013	2.59	13.67	0.86
Mckenna, Hugh [29,38,39]	3	59	2013	2.96	19.67	0.99
Batsis, John A. [35,40]	2	39	2017	3.22	19.50	1.61
Coburn, AF [26,32]	2	53	2003	2.03	26.50	1.02
Cross, Maddalena [36,37]	2	41	2013	2.59	20.50	1.29
Finlayson, Sarah [34,36]	2	19	2012	1.20	9.50	0.60
Gessert, Charles E. [30,43]	2	38	2007	1.13	19.00	0.56
Haller, Irina V. [25,30]	2	38	2007	1.13	19.00	0.56
Jia, Jihui [21,22]	2	51	2018	4.80	25.50	2.40
Kane, Robert L. [4,30]	2	78	2010	2.68	39.00	1.34
Kivela, SL [41,42]	2	92	1996	2.00	46.00	1.00
Li, Yuqin [22,24]	2	51	2018	4.80	25.50	2.40
Meng, Hongdao [23,33]	2	56	2012	2.19	28.00	1.09
Ryan, Assumpta [38,39]	2	24	2013	1.21	12.00	0.61
Su, Yonggang [21,22]	2	51	2018	4.80	25.50	2.40
Sun, Yaoyao [21,22]	2	51	2018	4.80	25.50	2.40
Wu, Menglian [21,22]	2	51	2018	4.80	25.50	2.40
Xie, Hui [21,22]	2	51	2018	4.80	25.50	2.40
Yang, Yang [21,22]	2	51	2018	4.80	25.50	2.40
Zhang, Dan [21,22]	2	51	2018	4.80	25.50	2.40
Zhao, Xia [22,31]	2	51	2018	4.80	25.50	2.40

Note: The reference citation number herein is the reference citation number in the text.

**Table 3:** Table of countries actively engaged in research on rural institutional elder care.

Country	Number of Publications	Total Link Strength	Avg. Pub. Year	Norm. Citations	Ave. Citation	Ave. Norm. Citation
USA	58	8	2012	59.69	22.43	1.03
Australia	13	5	2011	13.42	15.38	1.03
China	8	3	2017	10.77	25.50	1.35
Germany	5	7	2015	4.69	4.60	0.94
Norway	5	4	2014	2.74	11.60	0.55
Sweden	5	8	2017	6.29	11.20	1.26
Canada	4	0	2014	4.36	15.75	1.09
Japan	4	1	2015	6.28	17.25	1.57
North Ireland	4	2	2014	3.66	17.50	0.91
Finland	2	0	1996	2.00	46.00	1.00
France	2	0	2016	0.22	1.50	0.11
Netherlands	2	4	2019	0.99	4.00	0.49
South Korea	2	1	2016	1.16	6.00	0.58
Czech Republic	1	4	2020	0.42	2.00	0.42
England	1	2	2014	0.69	11.00	0.69
Israel	1	4	2020	0.42	2.00	0.42
Italy	1	4	2020	0.42	2.00	0.42
Poland	1	0	2018	0.28	3.00	0.28
Portugal	1	0	2021	1.80	1.00	1.80
Scotland	1	2	2014	0.69	11.00	0.69
Singapore	1	1	2011	0.16	4.00	0.16
Slovenia	1	2	2018	0.47	5.00	0.47
Switzerland	1	0	2002	1.45	56.00	1.45

**Table 4:** Summaries of main keywords in the rural institutional elder care research.

Keywords	Occurrence	Avg. Pub. Year	Ave. Citation	Ave. Norm. Citation
nursing home	44	2015	15.70	1.09
of-care	28	2014	15.89	0.87
long-term care	19	2014	21.53	1.00
risk factors	19	2010	30.37	1.11
health	17	2012	15.59	0.76
dementia	16	2014	17.75	1.04
older-adults	15	2014	17.93	0.99
rural	14	2014	14.79	0.82
quality of care	11	2015	16.45	0.95
mortality	10	2014	16.90	0.98
depression	8	2012	19.50	1.14
hospitalization	8	2013	13.13	0.64
nursing-home residents	8	2011	29.38	0.96
older adults	8	2016	15.13	1.47
services	8	2011	14.63	0.82
community	7	2010	11.71	1.23
outcomes	7	2011	23.43	1.24
healthcare	6	2012	22.17	0.93
impact	6	2020	2.50	0.93
institutionalization	6	2005	31.67	1.08
staff	6	2013	17.33	1.16
urban	6	2010	19.83	0.63
aged care	5	2012	10.60	0.65
cognitive impairment	5	2015	37.60	1.31
older adults	5	2007	19.20	0.55
predictors	5	2012	30.00	0.93
adults	4	2017	13.25	0.79
disparities	4	2019	13.00	1.28
experiences	4	2015	5.75	0.45
facility residents	4	2015	14.50	0.93
management	4	2012	16.50	0.87
medicare	4	2018	15.75	1.31
mini-mental-state	4	2010	13.75	0.62
prevalence	4	2016	12.75	0.70
systems	4	2012	15.75	0.51

## Data Availability

Not applicable.
